# Secondary trauma response in emergency services systems (STRESS) project: quantifying and predicting vicarious trauma in emergency medical services personnel

**DOI:** 10.29045/14784726.2023.3.7.4.23

**Published:** 2023-03-01

**Authors:** Ginny K. Renkiewicz, Michael W. Hubble

**Affiliations:** Rush University, Chicago, IL; Methodist University, Fayetteville, NC; Wake Technical Community College, Raleigh, NC

**Keywords:** emotional countertransference, paramedic, traumatic stress, vicarious trauma

## Abstract

**Introduction:**

There is a lack of literature exploring vicarious trauma (VT) in emergency medical services (EMS) personnel. VT is emotional countertransference that occurs between the clinician and patient. The presence of trauma- or stressor-related disorders could be a factor in the rising suicide rate in these clinicians.

**Methods:**

This was a cross-sectional statewide study of American EMS personnel, using one-stage area sampling. Nine EMS agencies were selected to participate based on geographic area, who then provided data about annual call volume and mix. The Impact of Event Scale-Revised was used to quantify VT. Univariate analyses used chi-square and ANOVA to evaluate the relationship between VT and various psychosocial and demographic characteristics. Factors significant in the univariate analyses were included in a logistic regression to determine predictors of VT while controlling for potential confounders.

**Results:**

A total of 691 respondents participated in the study, of which 44.4% were female and 12.3% were minorities. Overall, 40.9% had VT. Of those, 52.5% scored high enough to potentially illicit immune system modulation. Compared to those without VT, more than four times as many EMS professionals with VT self-reported as currently in counselling (9.2% v. 2.2%; p < 0.01). Approximately one in four EMS professionals (24.0%) had considered suicide, while nearly half (45.0%) knew an EMS provider who had died by suicide. There were multiple predictors of VT, including female sex (odds ratio [OR] 1.55; p = 0.02) and childhood exposure to emotional neglect (OR 2.28; p < 0.01) or domestic violence (OR 1.91; p = 0.05). Those with other stress syndromes, such as burnout or compassion fatigue, were 2.1 and 4.3 times more likely to have VT, respectively.

**Conclusions:**

Among study participants, 41% suffered from VT, and 24% had considered suicide. As a largely understudied phenomenon in EMS professionals, additional research on VT should focus on causality and the mitigation of sentinel events experienced in the workplace.

## Introduction

This paper is part of the secondary trauma response in emergency services systems (STRESS) project, which was designed to quantify trauma- and stressor-related disorders in emergency medical services (EMS) professionals and determine predictors in this unique population (Renkiewicz & Hubble, 2021).

There were 89 documented paramedic or firefighter suicides in the 120 years prior to the terrorist attacks of 9/11 (Busch, 2014). Alarmingly, this rate has exploded in the last 20 years, with an astounding 444 responder suicides – a relative 500% increase (Busch, 2014). Additionally, there is no centralised reporting system for such events, which means that the rates of suicidality in all public safety domains may actually be higher. Trauma- and stressor-related disorders, such as vicarious trauma (VT), have been described in the literature since 1990 and could play a considerable role in this phenomenon (Busch, 2014; Kadambi & Truscott, 2004; McCann & Pearlman, 1990). However, there is a scarcity of literature examining the prevalence and predictors of VT in EMS professionals.

VT is defined as a form of emotional countertransference that occurs between a clinician and their patient (Brown, 2011). It is described as the exposure to trauma (experienced either directly or indirectly) that causes maladaptive coping (Brown, 2011; Kadambi & Truscott, 2004; McCann & Pearlman, 1990). Some symptoms are subtle (e.g. irritability, difficulty sleeping, general dissatisfaction), while other symptoms are more overt and disruptive, such as hopelessness, withdrawal from colleagues, or questioning one’s worldview (Brown, 2011; Busch, 2014; Kadambi & Truscott, 2004; McCann & Pearlman, 1990; Pearlman & Mac Ian, 1995; Renkiewicz & Hubble, 2021). Clinicians suffering from VT are often transformed after experiencing or witnessing the trauma of others – a term known as critical incident stress, which is a type of traumatic stress (Baird & Jenkins, 2003; Baird & Kracen, 2006; Hudnall Stamm, 2012).

Individuals are reliant upon the mechanisms built throughout their upbringing to explain circumstances or events that occur in their lives (Helm, 2019; McCann & Pearlman, 1990). The violent presentation of trauma without an adequate coping structure can cause a coping maladaptation in the individual, which can ultimately produce and progress VT (Brown, 2011; McCann & Pearlman, 1990). These behavioural adaptations change an individual’s belief system and the way that individual experiences the world, and are both extensive and cumulative (Brown, 2011; McCann & Pearlman, 1990).

VT is often confused with other trauma- or stressor-related disorders, such as post-traumatic stress injury (PTSI), burnout or compassion fatigue. However, VT is a unique construct. Fundamentally, VT results from emotional contagion (Brady et al., 1999; Branson, 2019; Devilly et al., 2009; Hayes, 2013; Sansbury et al., 2015). A maladaptation occurs during the emotional trauma experienced during the caregiver–patient relationship. These alterations can be physical, spiritual, cognitive, neurological or emotional (Hayes, 2013; Sansbury et al., 2015). Essentially, VT explores the emotional impact of empathy during trauma. Alternatively, PTSI results from first-hand exposure to an event that causes anxiety (Sansbury et al., 2015). While these two trauma- or stressor-related disorders can have analogous symptomatology, PTSI is a first-hand experience, while VT is a second-hand experience. Burnout is resultant from a compromised working environment, which could include poor leadership or morale, lack of sleep, lack of administrative support, and so on (Hayes, 2013; Sansbury et al., 2015). There is also compassion fatigue that, like burnout, can produce similar psychopathology. However, it is the root of compassion fatigue that varies, in that it occurs due to a prolonged exposure to traumatic material (Figley, 1995; Renkiewicz & Hubble, 2021).

Cognitive and emotional systems are not the only ones that experience change in this environment; there are psychobiological changes as well. The body’s response to critical incident stress is known as an acute stress reaction, which is regulated by the hypothalamus-pituitary-adrenal (HPA) axis. An acute stress reaction is defined as the accumulation of stress that interrupts normal function and is usually short in duration, allowing the HPA axis to physiologically process the event and return the body to a homeostatic state (Chu et al., 2022). However, a prolonged acute stress reaction resulting from critical incident stress can result in post-traumatic stress injury (Chu et al., 2022; Halpern et al., 2011, 2012; Koren et al., 2002; Shalev et al., 1998) or other primary trauma- or stress-related disorders, such as VT, and can lead to HPA axis dysregulation (Beaton et al., 1999; Chu et al., 2022). HPA axis dysregulation can produce baseline hypercortisolism, cytokine reactions, and immune system suppression (Silverman et al., 2005). The psychobiological damage caused by trauma- or stressor-related disorders and HPA axis dysregulation is associated with a myriad of medical conditions, including atherosclerosis, osteoporosis, acute myocardial infarction and obesity (Chu et al., 2022; Silverman et al., 2005).

The purpose of this study was to determine the prevalence and predictors of VT among a statewide sample of American EMS personnel. It is possible that the presence of VT could be triggered by multiple factors, such as a history of childhood adversity, or a combination of demographic, employment, and socioeconomic factors. This study aims to quantify VT in EMS professionals and explore potential predictors, which might promote cultural change, advanced education, and additional support within, and for, the EMS profession.

## Methods

### Study design

Institutional Review Board approval for this cross-sectional study was obtained from Rush University. The state of North Carolina is divided into four geographic regions (mountains, piedmont, coastal plains, and tidewater) (Suburbanstats, 2016). EMS agencies covering county areas from 1012.01 to 2446.87 km^2^ from each geographic region were assessed for human settlement area (HSA) and labour region (North Carolina Department of Commerce, 2016; United States Census Bureau, 2010). The goal was to have each HSA represented in each geographic region and to have representation from all eight labour regions. Twelve agencies were selected and asked to participate; of those, nine consented. In total, four rural, three suburban, and two urban EMS agencies participated. The HSA of each agency was predetermined using United States Census data (Suburbanstats, 2016). Six of eight labour regions were represented, with only the Northwest and West regions unrecorded. A six-question agency information questionnaire was sent to EMS departmental leadership to determine agency-specific factors, such as annual call volume and agency type and call mix, which may contribute to the development of VT in EMS personnel through exposure to critical incident stress.

An a priori power analysis was conducted using G*Power 3.1.9.7 analysis software (Heinrich Hein University, Düsseldorf, Germany) to determine necessary sample size, and identified that a minimum of 587 participants would be required to detect a difference in Impact of Event Scale-Revised (IES-R) scores in EMS professionals with 90% power, a 4% margin of error, and an effect size of 0.4. Given the potential for non-completers, the estimated sample size was adjusted by 25%, indicating that a minimum of 734 EMS professionals would be required for this study.

EMS professionals were recruited in person between March and July 2019, during monthly continuing education evolutions where they were administered a 105-item paper survey designed to quantify the rates of trauma- or stressor-related disorders that could then be compared to various factors.

### Study setting and sample

The majority of North Carolina’s 415 EMS agencies are third service and do not provide dual-role fire response. Generally, paramedic-level fire departments are in small towns and provide mutual aid to the privately or county-operated EMS system as first responders; in essence, pre-hospital patient care is not a primary occupational function. Of the state’s 100 counties, paramedic-level care is available in all but two, which are served by advanced emergency medical technician (AEMT)-level EMS systems.

Included in this study were primary, 911-response EMS professionals (EMTs, AEMTs, and paramedics). Emergency medical responders, emergency medical dispatchers, rotor or fixed-wing flight clinicians, and emergency responders in fire-based, dual-agency response services were excluded. However, these populations were assessed as measures of secondary employment, particularly as such employment augmented a primary 911 employment role.

Employer-sponsored monthly continuing education is a systematised process in North Carolina where EMS professionals attend monthly sessions to earn hours for recredentialing according to state-proscribed guidelines (North Carolina Office of Emergency Medical Services, 2020). EMS professionals were recruited in person during their monthly continuing education sessions and were administered the survey, which took approximately 15–25 minutes to complete. Respondents were neither incentivised to participate nor penalised for refusal and were allowed to leave the room. Written informed consent was obtained prior to survey distribution. Because of the likelihood of evoking strong emotional responses from participants, every effort was made to provide resources for crisis intervention and counselling, including the contact information for a local licenced professional counselling associate who is also a 30-year veteran paramedic. These resources were provided on the last page of the survey, which respondents were encouraged to keep.

### Survey instrument

The survey contained 21 questions to construct a sociodemographic profile, while adversity in childhood was measured using the 10-item adverse childhood experiences (ACEs) questionnaire on childhood trauma (Beck et al., 2008; Dube et al., 2003; Murphy et al., 2014). The ACEs questionnaire has been used across many professions and populations and shows acceptable-to-good internal consistency (Cronbach’s α = 0.57−0.88). It contains 10 questions and was used to quantify the presence of traumatic events that had occurred in the respondent’s childhood. A score of 1 is provided for any affirmative answer, with 10 as the maximum score on the questionnaire (Beck et al., 2008; Dube et al., 2003; Murphy et al., 2014). The survey included a question on gender, since trauma- or stressor-related disorders and PTSI have been reported as higher in transgender populations (Progovac et al., 2020). Additional profiles were created for employment- and mental health-related factors. The 17-question Life Events Checklist for *DSM-5* was included to determine how respondents had experienced critical incident stress and at what frequency. Trauma- or stressor-related disorders were evaluated using validated psychological instruments: subscales on the Professional Quality of Life (ProQOL) scale were used to determine the likelihood of compassion fatigue and burnout, while the 22-question IES-R was used to determine the likelihood of VT (Brady et al., 1999; Pearlman & Mac Ian, 1995; Schauben & Frazier, 1995).

The IES-R is designed to assess the respondent’s distress over the preceding seven days, as it pertains to a specific event (Horowitz et al., 1979; Weiss & Marmar, 1997). The scale is measured on a 5-point Likert scale from 0 (not at all) to 4 (extremely). The highest possible score on the IES-R is 88, and the lowest is 0. Three subscales created based on the tripartite model of PTSI include hyperarousal-, avoidance-, and intrusion-based symptom clusters outlined by the *DSM-5* and related to the psychological processing of critical incident stress (Horowitz et al., 1979; Weiss & Marmar, 1997). The instrument has excellent internal consistency (Cronbach’s α = 0.84–0.94) and test–retest reliability (Pearson’s *r* = 0.89–0.94) (Beck et al., 2008; Weiss & Marmar, 1997).

### Statistical analysis

Surveys were coded and then abstracted data were compiled and analysed using IBM SPSS Statistics version 25 (IBM Corporation, Armonk, NY), with statistical significance established at p ≤ 0.05. Continuous variables were compared using unpaired t-test or ANOVA, while categorical data were analysed using the chi-square test where appropriate. Where required, non-parametric testing was utilised for non-normally distributed variables. Variables significant in the univariate analysis were used to construct a multivariable logistic regression model to determine predictors of VT after controlling for potential confounders.

A dummy variable (yes/no) was calculated for whether the respondent had VT using raw scores on the IES-R; this variable was used as the outcome variable. A score of ≥ 24 on the IES-R indicates that VT is of clinical concern, meaning that VT is very likely present and at minimum, partial symptomatology is observable (Asukai et al., 2002; Beck et al., 2008; Murphy et al., 2014; Weiss & Marmar, 1997). Scores ≥ 37 represent distress high enough to modulate immune system function (Asukai et al., 2002; Beck et al., 2008; Murphy et al., 2014; Shelton & Kelly, 1995; Weiss & Marmar, 1997).

## Results

A total of 811 EMS professionals were recruited to participate in the study, of which 770 consented. Of those, 706 individuals participated in the VT portion of the survey during the study period, and 691 completed the survey. This yielded a response rate of 87.1% and a completion rate of 85.2%. In all, 282 (40.8%) EMS professionals were identified as likely having VT.

### Sociodemographic profile

In comparing those with and without VT, statistically significant differences between the groups were observed with respect to sex, age and annual income. EMS professionals who were transgender were unable to be included due to small sample sizes. Differences in provider education level, marital status, sexual orientation and credential level were not statistically significant between the two groups (Table 1).

**Table 1.** Sociodemographic profile of EMS professionals with VT compared to the overall sample.

	With VT 282 (40.8%)		Without VT 409 (59.2%)		Overall n = 691		p-value
	n	%	n	%	n	%	
**Sex**							
Female	149	52.8	158	36.6	307	44.4	< 0.001
Male	133	47.2	251	61.4	384	55.6	
**Sexual orientation**							
Homosexual	20	7.1	25	6.1	45	6.6	0.259
Heterosexual	252	89.3	377	92.1	629	91.0	
Other	10	3.6	7	1.7	17	2.5	
**Education**							
High school diploma	21	7.4	35	8.6	56	8.1	0.116
Some college	106	37.6	133	32.5	239	34.6	
Associate degree	89	31.6	123	30.1	212	30.7	
Bachelor’s degree	54	19.1	107	26.2	161	23.3	
Master’s or doctorate	12	4.3	11	2.7	23	3.3	
**Minority status**	36	12.8	49	12.0	85	12.3	0.484
**Hispanic or Latinx**	8	2.9	14	3.5	22	3.2	0.826
**Marital status**							
Single	112	39.9	156	38.3	268	38.8	0.187
Married	121	42.7	195	47.4	316	45.7	
Separated	13	4.6	10	2.5	23	3.3	
Divorced or widowed	36	12.8	48	11.8	84	12.2	
**Annual income**							
< $20,000	6	2.1	16	3.9	22	3.2	0.007
$20,001–$40,000	86	30.5	91	22.2	177	25.6	
$40,001–$60,000	70	24.8	114	27.9	184	26.6	
$60,001–$80,000	63	22.3	80	19.6	143	20.7	
$80,001–$100,000	42	14.9	58	14.2	100	14.5	
> $100,000	15	5.3	50	12.2	65	9.4	
**Credential level**							
EMT	56	19.9	82	20.0	138	20.0	0.991
AEMT	20	7.1	28	6.8	48	6.9	
Paramedic	206	73.0	299	73.1	505	73.1	
**Continuous variables**	**Mean**	**SD**	**Mean**	**SD**	**Mean**	**SD**	**p-value**
Age	33.41	9.55	35.15	10.47	34.44	10.13	0.028

AEMT: advanced emergency medical technician; EMS: emergency medical services; EMT: emergency medical technician; SD: standard deviation; VT: vicarious trauma.

### Employment profile

A full employment profile of EMS professionals with and without VT can be found in Table 2. All nine agencies included in the study primarily provided 911 response, and 84.3% of employees worked in an agency that provided mobile integrated healthcare as a secondary service. Comparing those with and without VT, differences in years of field experience and shift length were statistically significant, and on average, shifts were 1.5 hours longer in those with VT. The HSA where the EMS professional provided service was not significant. However, in terms of exposure to potentially traumatic experiences, the numbers of calls related to intimate partner violence, paediatric death, electrocution and/or burns, obvious death, and mass casualty incidents were all significantly different between groups.

**Table 2.** Employment profile of EMS professionals.

	With VT 282 (40.8%)		Without VT 409 (59.2%)		Overall n = 691		
Categorical variables	n	%	n	%	N	%	p-value
**HSA**							
Rural	109	37.9	149	36.4	256	37	0.362
Suburban	117	41.5	157	38.4	274	39.7	
Urban	58	20.6	103	25.2	161	23.3	
**Employment status (full time)**	234	83	318	77.8	552	79.9	0.055
**Primary role (patient attendant)**	243	86.2	360	88	603	87.3	0.741
**Secondary employment**							
EMS	126	44.7	160	39.2	286	41.4	0.088
Fire service	39	13.9	71	17.4	110	16	0.246
Law enforcement	6	2.1	12	2.9	18	2.6	
Other public safety	23	8.2	21	5.1	44	6.4	
None	214	75.8	303	74.5	517	75	
**Years of field experience**							
< 1	15	5.3	33	8.1	48	6.9	0.050
1–5	77	27.3	122	29.9	199	28.8	
6–10	89	31.6	99	24.2	188	27.2	
11–15	41	14.5	45	11	86	12.4	
16–20	23	8.2	34	8.1	57	8.2	
21–25	20	7.1	33	8.1	53	7.7	
> 25	17	6	43	10.5	60	8.7	
**Agency type**							
Municipal (government)	239	84.8	334	81.7	573	82.9	0.305
Private, hospital-based	43	15.2	75	18.3	118	17.1	
**Agency response (primary 911)**	282	100	409	100	691	100	
**Secondary agency response**							
N/A; no secondary services	36	12.8	38	9.3	74	10.7	0.162
Convalescent transport	–	–	–	–	–	–	
Critical care	17	6.00	17	4.2	34	4.9	
MIH	229	81.2	354	86.6	583	84.4	
HEMS	–	–	–	–	–	–	
**Continuous variables**	**Mean**	**SD**	**Mean**	**SD**	**Mean**	**SD**	**p-value**
Shift length (in hours)	16.62	6.29	15.13	5.67	15.74	5.97	0.001
Annual call volume	37,508.70	25,391.89	43,069.36	25,584.13	40,800.03	25,633.73	0.005
OHCA	340.04	191.92	351.66	200.48	346.92	196.98	0.446
IPV	8.91	7.69	10.58	8.15	9.86	7.99	0.014
Death of a child	12.43	11.08	14.61	11.59	13.72	11.43	0.014
Electrocution and/or burns	46.27	26.34	50.43	27.34	48.74	26.99	0.046
Multi-system trauma	406.08	823.15	388.85	783.83.	395.88	799.56	0.781
Homicide	17.63	10.21	16.25	10.23	16.94	10.23	0.259
Suicide	43.94	28.72	38.40	30.69	40.96	29.89	0.061
Psychiatric calls	1422.10	1096.94	1468.51	1188.61	1449.57	1151.49	0.603
High-risk childbirth	67.68	104.60	82.35	122.48	76.07	115.29	0.145
Familial or domestic violence	85.86	62.96	92.01	71.45	89.38	67.95	0.300
Obvious death	372.09	555.09	476.60	605.15	433.95	587.08	0.021
MCI	88.89	142.10	121.70	153.34	107.46	149.33	0.008

EMS: emergency medical services; HSA: human settlement area; HEMS: helicopter emergency medical services; IPV: intimate partner violence; MCI: mass casualty incident; MIH: mobile integrated healthcare; OHCA: out-of-hospital cardiac arrest; SD: standard deviation; VT: vicarious trauma.

### Childhood trauma profile

The ACEs questionnaire was used to evaluate whether a history of adversity in childhood would be significant when compared to the presence of VT. Cronbach’s alpha for this study was 0.78, representing good internal consistency, which was congruent with previous studies (Dube et al., 2003; Murphy et al., 2014). Of the 10 questions on the ACEs questionnaire, all were statistically significant between those with and without VT, except for parental divorce and whether the respondent had experienced familial incarceration. The proportion in most categories was 1.5–2.7 times higher in EMS professionals with VT compared to those without. On average, the mean score on the ACEs was over a full point higher in EMS professionals with VT compared to those without (2.76 ± 2.56 v. 1.70 ± 1.85; p < 0.01). Results for the childhood trauma profile can be found in Table 3.

**Table 3.** Childhood trauma profile of EMS professionals (as determined by answers on the ACEs questionnaire).

	With VT 282 (40.8%)		Without VT 409 (59.2%)		Overall n = 691		
	n	%	n	%	n	%	p-value
Physical abuse	81	28.7	78	19.1	159	23.0	0.003
Emotional abuse	113	40.1	103	25.2	216	31.3	< 0.001
Sexual abuse	55	19.5	44	10.8	99	14.3	0.002
Physical neglect	28	9.9	15	3.7	43	6.2	0.001
Emotional neglect	89	31.6	49	12.0	138	20.0	< 0.001
Divorce	134	47.5	175	42.8	309	44.7	0.243
Domestic violence	45	16.0	27	6.6	72	10.4	< 0.001
Substance abuse in the home	103	36.5	96	23.5	199	28.8	< 0.001
Familial depression, mental illness, or suicide	93	33.0	82	20.0	175	25.3	< 0.001
Incarcerated family member	25	8.9	25	6.1	50	7.2	0.181
**Continuous variables**	**Mean**	**SD**	**Mean**	**SD**	**Mean**	**SD**	**p-value**
ACEs total score	2.76	2.56	1.70	1.85	2.11	2.23	< 0.001

ACEs: adverse childhood experiences questionnaire; EMS: emergency medical services; SD: standard deviation; VT: vicarious trauma.

### Mental health profile

EMS professionals completed survey questions related to their understanding of what agency resources were available to them if they experienced a sentinel event (Table 4). There were no differences between groups. For EMS professionals with VT, 1.6 times as many had sought counselling for a stress-related event, while over four times as many identified as currently being in counselling (9.2% v. 2.2%, p < 0.01).

**Table 4.** Mental health profile of EMS professionals.

	With VT 282 (40.8%)		Without VT 409 (59.2%)		Overall n = 691		
	n	%	n	%	n	%	p-value
**Awareness of agency resources**							
Incident debriefing	225	79.8	341	83.6	566	82.0	0.226
Departmental chaplain	26	9.3	50	12.3	76	11.0	0.265
CIT	161	57.1	236	57.8	397	57.5	0.876
EAP	168	59.6	265	65.0	433	62.8	0.173
Exercise programmes	24	8.5	30	7.4	54	7.8	0.667
Time off	95	33.7	136	33.3	231	33.5	0.935
**Prior history of counselling for a stress-related event**	117	41.5	109	26.7	226	32.7	< 0.001
**Currently in counselling**	26	9.2	9	2.2	35	5.1	< 0.001
**Likelihood of having BO**	212	75.2	185	45.2	397	57.5	< 0.001
**Likelihood of having CF**	208	73.8	124	30.3	332	48.0	< 0.001
**Has considered suicide**	98	34.8	68	16.6	166	24.0	< 0.001
**Knows an EMS professional who died from suicide**	134	47.5	177	43.3	311	45.0	0.277

BO: burnout; CF: compassion fatigue; CIT: crisis intervention team; EAP: employee assistance programme; EMS: emergency medical services; VT: vicarious trauma.

Overall, roughly one in four EMS professionals surveyed had a lifetime prevalence of suicide (24%, n = 166), and the lifetime prevalence of suicidality in EMS professionals with VT was more than double the prevalence of those without VT (34.8% v. 16.6%, p < 0.01). Altogether, 45% (n = 311) of respondents knew an EMS professional who had died by suicide.

Of particular interest is the proportion of respondents who were likely to have a concomitant stress condition, such as burnout or compassion fatigue. In those with VT, those proportions were 75.2% (n = 212) and 73.8% (n = 208) for burnout and compassion fatigue, respectively, compared to just 45.2% (n = 185) and 30.3% (n = 124) in those without VT.

### Severity of vicarious trauma in emergency medical services professionals

Cronbach’s alpha for the IES-R was 0.96, representing excellent internal consistency. Of the 282 (40.8%) respondents who were identified as having VT on the IES-R, 146 (52.4%) had scores ≥ 37, which is reportedly significant enough to modulate and suppress immune system function (Kawamura et al., 2001; Silverman et al., 2005). The mean (± SD) score for those with and without VT was 41.01 (± 14.05) versus 8.13 (± 7.28), respectively. The median score for those with VT was 38 (IQR = 30–49), while for those without VT it was 6 (IQR = 1–14). The highest recorded score was 86, which was just below the maximum attainable score of 88.

Mean scores for each subscale were five to seven times higher in participants identified as having VT. The severity profile of EMS professionals with VT is outlined in Table 5.

**Table 5.** Severity profile of vicarious trauma in EMS professionals.

**Categorical variables**						n	%
Exhibiting some symptoms (total scores 24–32)						85	30.1
VT is of clinical concern and symptoms are present (total scores 33–36)						49	17.4
VT with additional physical manifestations, including immune-system suppression (total scores ≥ 37)						148	52.4
	**With VT** **282 (40.8%)**		**Without VT** **409 (59.2%)**		**Overall** **n = 691**		
**Continuous variables**	**Mean**	**SD**	**Mean**	**SD**	**Mean**	**SD**	**p-value**
IES-R total score	41.01	14.05	8.13	7.28	21.55	19.32	< 0.001
Intrusion subscale	1.97	0.77	0.41	0.43	1.05	0.97	< 0.001
Avoidance subscale	1.97	0.71	0.43	0.47	1.06	0.95	< 0.001
Hyperarousal subscale	1.58	0.89	0.23	0.29	0.78	0.90	< 0.001

EMS: emergency medical services; IES-R: Impact of Event Scale-Revised; SD: standard deviation; VT: vicarious trauma.

### Predictors of vicarious trauma

A logistic regression was conducted to determine factors associated with VT in EMS professionals (Nagelkerke *R^2^* = 0.35). Variables significant in the univariate analysis were included in the multivariable analysis. Females were 55% more likely to have VT (odds ratio [OR] 1.55; 95% CI 1.05–2.29). EMS professionals who suffered emotional neglect in childhood were 2.28 (OR 2.28; 95% CI 1.33–3.89) times more likely to have VT, and those with a childhood history of domestic violence were 91% more likely to have VT (OR 1.91; 95% CI 1.01–3.78). EMS professionals who were identified as having other trauma- or stressor-related disorders, such as compassion fatigue and burnout, were 4.27 and 2.13 times more likely to have VT, respectively. Several annual income ranges were also predictive; respondents were nearly 4.5 times more likely to have VT if their annual income was between $40,000 and $60,000 (Table 6).

**Table 6.** Predictors of VT in EMS personnel.

	OR	95% CI	p-value
**Sociodemographic factors**			
Female sex	1.55	1.05–2.29	0.024
Has CF	4.27	2.90–6.27	< 0.001
Has BO	2.13	1.43–3.18	< 0.001
Previously engaged in counselling	1.29	0.40–4.14	0.671
Currently in counselling	0.92	0.27–3.09	0.889
Considered suicide	1.46	0.93–2.28	0.097
Field experience (> 5 years)	1.50	0.94–2.41	0.090
**Childhood trauma**			
Physical abuse	1.15	0.66–2.03	0.620
Emotional abuse	1.02	0.59–1.77	0.942
Sexual abuse	1.34	0.76–2.33	0.310
Physical neglect	0.94	0.41–2.17	0.887
Emotional neglect	2.28	1.33–3.89	0.003
Domestic violence	1.91	1.01–3.78	0.045
Substance abuse in the home	0.96	0.60–1.54	0.987
Familial mental illness, depression, or suicide	1.25	0.78–1.99	0.358
**Annual income level (< $20,000 = referent)**			
$20,000–$40,000	1.69	0.48–6.03	0.416
$40,001–$60,000	3.92	1.75–8.75	< 0.001
$60,001–$80,000	2.09	0.98–4.47	0.057
$80,001–$100,000	2.81	1.30–6.04	0.008
> $100,000	2.67	1.19–6.03	0.018

BO: burnout; CF: compassion fatigue; CI: confidence interval; EMS: emergency medical services; OR: odds ratio; VT: vicarious trauma.

## Discussion

In this study of 691 EMS personnel, we found that 40.8% had VT and nearly half knew an EMS professional who had died from suicide. Maladaptive coping and processing by EMS professionals may be part of the professional culture and could be the result of the self- or culturally imposed ‘EMS super-human’ myth described by Shelton and Kelly (1995, p. 30):

I can do it all. I’m always in control. Nothing fazes me, nothing bothers me, nothing shakes me. I divide my life into compartments. What happens out in the streets, in the field, doesn’t touch the other parts of my life. I’m immune. I don’t burden my family or friends with it – they wouldn’t understand anyway. I don’t cry, ever. Tears mean weakness and I’m strong. Nobody will see that I am sometimes upset or sometimes afraid. Nobody will see my fears. Nobody – nobody will call me soft.

Such internal and external pressures could create a confluence of experiences that prime an EMS professional to be more at risk for trauma- or stressor-related disorders, such as VT.

### The employment environment

It could be posited that HSA is a mitigating factor to the presence of VT in EMS professionals. Indeed, it could be hypothesised that higher call volumes would produce a higher exposure risk for critical incident stress that produces VT. However, those results were neither statistically significant nor predictive in our cohort. While no literature was found related to EMS professionals, several studies indicated that exposure and subsequent diagnosis of psychological trauma was higher in urban areas than in suburban or rural counterparts (McCall-Hosenfeld et al., 2014; Spinazzola et al., 2005). While HSA was not significant, many of the continuous variables related to the number of sentinel events collected by each participating agency were significant. As such, these numbers serve as a bellwether to indicate that it is not the volume of calls that creates the exposure risk, but the exposure to sentinel events within the agency, irrespective of HSA. This lends credence to the notion that it is not the volume of events that precipitates the development of VT but the nature of the event itself.

Nearly a quarter of the sample had secondary employment in public safety while working full time; however, the simple presence of secondary employment was not significant. For VT, this further serves to illustrate the notion that exposure risk may not be the volume of events that precipitates the development of VT but the exposure to specific sentinel events, regardless of cumulative time spent in the field.

### Impact of adverse childhood experiences

A score of ≥ 4 on the questionnaire has been considered a ‘high’ exposure to trauma in childhood that is directly correlated with adverse adult health (Dube et al., 2003). Such complications include obesity, cardiac disease, diabetes and decreased life expectancy (Dube et al., 2003). In this sample of EMS personnel, 181 (23.57%) scored ≥ 4 on the questionnaire, while the mean score was nearly a full point higher in those with VT compared to those without (2.76 ± 2.59 v. 1.68 ± 1.85, p < 0.001).

Emotional neglect and domestic violence experienced in childhood were predictive of VT, which is consistent with several studies on other types of healthcare providers (Vigil et al., 2018). Indeed, it could be postulated that parental failure to provide a nurturing environment in childhood where appropriate emotional processing was demonstrated might yield emotional overcompensation in the EMS professional, thus maximising susceptibility to emotional countertransference.

### Mental health and suicidality

Respondents with and without VT were equally aware of resources available to them at their agency when they had experienced a sentinel event. More than 1.5 times as many EMS professionals had sought counselling for a prior stress-related event, while 4.2 times as many identified as currently being in counselling. The prevalence of suicidality in EMS professionals with VT was double that of those without VT. These results were consistent with a Canadian study by Carleton et al. (2018) that compared suicidality in EMS professionals to that of the civilian population. This study showed that the prevalence of suicidality was 41.1% in paramedics – nearly double that of any other public safety profession, including military servicemembers (Carleton et al., 2018). However, Carleton et al.’s study did not differentiate the prevalence of suicidality by the presence or type of stress syndrome.

A recent study by Vigil et al. (2018) showed that 5.5% of EMTs had died by suicide – a crude mortality odds ratio 2.43 times higher than civilian rates in the same dataset. A study of adolescents indicated that prior exposure to suicide in others was a risk factor for future suicidality – a phenomenon known as suicide contagion (Conrad, 1992). These results were particularly relevant considering that our overall dataset showed a lifetime prevalence of suicidality in EMS professionals that was 24%, while nearly half knew another EMS professional who had died from suicide. However, despite the univariate significance of lifetime suicidality prevalence, it was not significant in the multivariable model. This is likely the result of trauma- or stressor-related disorders serving as risk factors for suicidality, not the other way round.

### Severity of vicarious trauma in emergency medical services professionals

Nearly 52% of EMS professionals had VT scores that have been associated in previous reports with significant HPA axis dysregulation (Kawamura et al., 2001; Silverman et al., 2005; Weiss & Marmar, 1997). Such dysfunction can result in hypercortisolism and the release of inflammatory cytokines, including changes in lymphocyte and T-cell counts (Kawamura et al., 2001; Weiss & Marmar, 1997). More importantly, studies on civilians with PTSI have shown that cellular immunity has been affected for up to 10 years after a single significant, sentinel event (Kawamura et al., 2001; Weiss & Marmar, 1997). The nature of EMS work produces the potential for repeated exposure to sentinel events with limited downtime, an environment that has been shown in studies of physicians and other healthcare providers to be detrimental to psychological, psychobiological and physical health (Brown, 2011; Kadambi & Truscott, 2004; McCann & Pearlman, 1990; Sullivan, 2012).

### Limitations

This study has several limitations, one of which is the generalisability of the findings to other settings. Web-based surveys traditionally have very low rates of return, and the size of this instrument made the online collection of the required sample size unlikely. To mitigate this limitation, this survey was conducted in person on a diverse sample of EMS personnel with an overall response rate of nearly 90%. However, the sample was derived from a single state, and the generalisability of our findings to EMS personnel outside of North Carolina is unknown.

Given the commonly insidious nature of traumatic stress syndrome symptomatology, some EMS professionals who were actually affected by such syndromes, may have refused to participate. There is some supposition that there is a relationship between HPA axis dysregulation and anosognosia, which is impaired insight due to an underlying physical condition (Kawamura et al., 2001; Silverman et al., 2005). Such lack of insight may have resulted in respondents who chose not to participate but were otherwise affected by VT, particularly when considering that a relationship has been shown between anosognosia and individuals with trauma- or stressor-related disorders (Kawamura et al., 2001).

An additional limitation is that this study only identified factors associated with VT and was cross-sectional, which does not allow for any statements related to causality.

Lastly, several of the EMS agencies did not have a mechanism to track sentinel events that occurred within the agency’s service area. Some agencies were unable to provide data on certain types of events, which may have affected the univariate analyses between the presence of VT and event type.

## Conclusions

To the best of our knowledge, this study is the first of its kind to quantify VT in EMS personnel, and more research is needed in this area. Nearly 41% of EMS professionals surveyed had VT; of those, 52.4% had scores identified by previous publications high enough to suggest the potential for HPA axis dysregulation and immune system dysfunction, a proportion of which could produce stimulation of the immune system for the following decade. Additional research should focus on the relationship between HPA axis dysregulation and anosognosia in traumatic stress. Those EMS professionals with concomitant stress syndromes, such as burnout and compassion fatigue, were 2.3 and 4.7 times more likely to also have VT, respectively. Nearly one in four participants reported suicidality, and nearly half knew an EMS provider who had died from suicide.

VT appears to be a significant problem in EMS professionals, and additional research should focus on causality, the mitigation of sentinel events in the workplace, and the identification and understanding of the role that childhood trauma plays in the daily experiences of EMS professionals.

These results may prove useful in promoting the cultural change necessary to support EMS professionals when they develop VT or other trauma- or stressor-related disorders, providing advanced education to inform EMS professionals and their peers about the signs and risks, and affording additional support to those who serve in the emergency medical services.

## Acknowledgements

The authors would like to acknowledge participating EMS agencies for their contribution to this project.

## Author contributions

GKR conceived the study concept, recruited participants, collected and coded the data, ran the statistical analysis, and wrote the first draft. MWH assisted with the statistical analysis and subsequent edits of the manuscript. GKR acts as the guarantor for this article.

## Conflict of interest

None declared.

## Ethics

This project underwent full review through the Rush University Institutional Review Board (ref: 18022001-IRB01).

## Funding

None.
